# Supplementary material for: Biological Control of Fruit Rot and Anthracnose of Postharvest Mango by Antagonistic Yeasts from Economic Crops Leaves
Source: Microorganisms. 2020 Feb 25;8(3):317. doi: 10.3390/microorganisms8030317 (PMC7143844; doi:10.3390/microorganisms8030317)
Supplement: Supplementary file 1 [file microorganisms-08-00317-s001.pdf]

Table S1 Yeast species and strains belonging to phylum Ascomycota and phylum Basidiomycota

Taxa	Strain	No. of strain
**Phylum Ascomycota, Subphylum Saccharomycotina**		
Candida citri	DMKU-RE05	1
Candida diddensiae	DMKU-RP155	1
Candida fermentati	DMKU-CP79	1
Candida floricola	DMKU-CP149	1
Candida fukuyamaensis	DMKU-CP312	1
Candida intermidia	YE-20	1
Candida maltose	DMKU-RP16	1
Candida metapsilosis	DMKU-RE07, DMKU-RE15, DMKU-RE18, DMKU-RE35	4
Candida michaelii	YE239	1
Candida nivariensis	DMKU-CE15, DMKU-CE18	2
Candida parapsilosis	DMKU-RP300, DMKU-SP64, DMKU-SP434	3
Candida pseudointermedia	DMKU-CE37, DMKU-CE59, DMKU-CE87, DMKU-CE142	4
Candida saopaulonensis	DMKU-CP708	1
Candida sp.	DMKU-CE01	1
Candida tropicalis	DMKU-RE48, DMKU-RE68, DMKU-RP14, DMKU-CE04, DMKU-SE-73, DMKU-SE80, DMKU-SE98	7
Candida uthaithanina	DMKU-CP825	1
Candida wangnamhkiaoensi	YE-166	1
Cyberlindnera minuta	DMKU-CP557, DMKU-SE74	2
Cyberlindnera rhodanensis	DMKU-RE125, DMKU-SE04, DMKU-SE22, DMKU-SE29, DMKU-SE84	5
Debaryomyces nepalensis	DMKU-RP03	1
Hyphopichia burtonii	YE-164	1
Kluyveromyces marianus	DMKU-CE134	1
Kodamaea ohmeri	DMKU-RE27, DMKU-RP06, DMKU-RP34, DMKU-RP233, DMKU-CP51, DMKU-CP471, DMKU-CP258, DMKU-SP45	8
Kodamaea sp.	DMKU-SP81	1
Metschnikowia lopburiensis	DMKU-CP680	1
Meyerozyma caribbica	DMKU-RE37, DMKU-RE75, DMKU-RE87, DMKU-RE95, DMKU-RE102, DMKU-RP07, DMKU-RP47, DMKU-CE27, DMKU-CE32, DMKU-CE33, DMKU-CE35, DMKU-CE38, DMKU-CE51, DMKU-CE56, DMKU-CE77, DMKU-CE83, DMKU-CE119, DMKU-CE123, DMKU-CE140, DMKU-CE629, DMKU-CE783,	46

Taxa	Strain	No. of strain
	DMKU-CE503, DMKU-CE666, DMKU-CE813, DMKU-SE01, DMKU-SE10, DMKU-SE12, DMKU-SE12, DMKU-SE13, DMKU-SE28, DMKU-SE42, DMKU-SE46, DMKU-SE65, DMKU-SE76, DMKU-SE78, DMKU-SE86, DMKU-SE87, DMKU-SE93, DMKU-SE96, DMKU-SE97, DMKU-SE101, DMKU-SP01, DMKU-SP24, DMKU-SP121, DMKU-SP197, DMKU-SP461	
Meyerozyma guilliermondii	DMKU-RP26	1
Pichia aff. fermentans	DMKU-CP733	1
Pichia kudriavzevii	DMKU-CE48, DMKU-CP672, DMKU-SE109, DMKU-SE114, DMKU-SE117, DMKU-SE140, DMKU-SE144, DMKU-SE145	8
Pichia myanmerensis	DMKU-CP657	1
Torulaspora indica	DMKU-RP31, DMKU-RP35	2
Wickerhamomyces anomalus	DMKU-RE13, DMKU-RE38, DMKU-RE62, DMKU-RP04, DMKU-RP25, DMKU-CP122, DMKU-CP571, DMKU-CE52, DMKU-CE55, DMKU-SE112, DMKU-SE132, DMKU-SP335	12
Yamadazyma epiphylla	YE-170	1
**Phylum Basidiomycota, Subphylum Agaricomycotina**		
Dioszegia zsoltii	DMKU-SP116	1
Kwoniella haveanensis	DMKU-CE102, DMKU-SP237	2
Hannella lutiola	DMKU-CP282	1
Hannella oryzae	DMKU-CP174, YE-69, DMKU-CP775	3
Hannella pagnoccae	DMKU-CP636, DMKU-SP56	2
Hannella phyllophila	DMKU-CP648, DMKU-CP673, DMKU-SP186	3
Hannella sinensis	DMKU-CP437, DMKU-CP606, DMKU-CP665, DMKU-CP712, DMKU-CP181, DMKU-CP321, DMKU-SE105, DMKU-SP222, YE-56, YE-58	10
Hannella siamensis	DMKU-CP09, DMKU-CP631, YE-124	3
Naganishia albidosimilis	DMKU-CE74, DMKU-CE85	2
Naganishia liquefaciens	DMKU-CE05	1
Papiliotrema aspenensis	DMKU-CE31, DMKU-SP67, DMKU-SP423, YE-105	4
Papiliotrema flavescens	DMKU-CE11, DMKU-CE22, DMKU-CE129, DMKU-CE139, DMKU-CP654, DMKU-CP734, DMKU-SE92, DMKU-SP20, DMKU-SP231, YE-26, YE-127	11

Taxa	Strain	No. of strain
Papiliotrema japonica	DMKU-RE36, DMKU-SE03, DMKU-SE69, YE-135	4
Papiliotrema laurentii	DMKU-CE109, DMKU-CP560, DMKU-CP585, YE-23	4
Papiliotrema nemorosus	DMKU-RP276, DMKU-CP38, DMKU-CP391	3
Papiliotrema rajasthanensis	DMKU-CP10, DMKU-CP603, DMKU-CP823, DMKU-SP89	4
Papiliotrema ruineniae	DMKU-CP781, DMKU-CP818	2
Papiliotrema siamense	DMKU-RP152, DMKU-SP199, DMKU-SE34	3
Papiliotrema sp.	DMKU-RE45, DMKU-RE85, DMKU-CE70, DMKU-CE95, DMKU-CP115, DMKU-SE32, DMKU-SE67, DMKU-SE107	8
Piskurozyma taiwanensis	DMKU-SP98	1
Saitozyma flava	DMKU-RE19, DMKU-RE30, DMKU-CP150, DMKU-SE94, DMKU-SP117, DMKU-SP285	6
Saitozyma sp.	DMKU-SE48, DMKU-SE56	2
Trichosporon asteroides	YE-169	1
Vishniacozyma taibaiensis	YE-155	1
**Phylum Basidiomycota, Subphylum Pucciniomycotina**		
Cystobasidium sp.	DMKU-RE72	1
Occultifer plantarum	DMKU-RP30	1
Occultifur sp.	DMKU-SE45, DMKU-SE129	2
Occultifur tropicalis	DMKU-SE38, DMKU-SE59	2
Rhodosporiobolus fluvialis	DMKU-CP293	1
Rhodosporiobolus ruineniae	DMKU-CE136	1
Rhodotorula mucilaginosa	DMKU-RE113, DMKU-RP68, DMKU-CE05, DMKU-CE30, DMKU-CE91, DMKU-CE110, DMKU-CP165, DMKU-CP596, DMKU-SE81, DMKU-SE91, DMKU-SP02	11
Rhodotorula paludigena	DMKU-RP198, DMKU-CE132, DMKU-CP170, DMKU-CP218, DMKU-CP625, DMKU-CP752, , DMKU-SP381, YE-25	8
Rhodotorula slooffiae	DMKU-CP485	1
Rhodotorula sp.	DMKU-RE31, DMKU-CE121, DMKU-CP209, DMKU-SE27	4
Rhodotorula taiwanensis	DMKU-RE57, DMKU-RE64, DMKU-RE96, DMKU-RE106, DMKU-RE115, DMKU-RP252, DMKU-CE03, DMKU-CE49, DMKU-CE53, DMKU-CE61, DMKU-CE78, DMKU-CE80, DMKU-CE88, DMKU-CE94, DMKU-CE106,	20

**Taxa**	**Strain**	**No. of strain**
	DMKU-CE118, DMKU-CE126, DMKU-CP18, DMKU-SP35, DMKU-SP63	
Rhodotorula toruloides	DMKU-RP49, DMKU-CP570	2
Sakaguchia oryzae	DMKU-RP190	1
Sporidiobolus carnicolor	DMKU-SP75, DMKU-SP181	2
Sporidiobolus pararoseus	DMKU-RE112, DMKU-RP134, DMKU-CP183, DMKU-CP195, DMKU-CP305, DMKU-CP799, DMKU-SE11	7
Sporobolomyces blumeae	DMKU-CP601, DMKU-CP613, DMKU-SP12, DMKU-SP47, YE15	5
Sporobolomyces nakasei	DMKU-CP741	1
Sporobolomyces carnicolor	DMKU-RE14, DMKU-RE33, DMKU-RE44, DMKU-RE47	4
Sporobolomyces sp.	DMKU-RE104	1
Symmetrospora marina	DMKU-SP251, DMKU-SP281	2
Symmetrospora vermiculata	DMKU-RP33, DMKU-SP261, DMKU-SP307	3
**Phylum Basidiomycota, Subphylum Ustilaginomycotina**		
Dirkmeia churashimeansis	DMKU-RP27, DMKU-CP612, DMKU-CP692, DMKU-SP49, DMKU-SP97	5
Jaminaea angkorensis	DMKU-SP36, DMKU-SP416	2
Moesziomyces aphidis	DMKU-CP111, DMKU-CP220, DMKU-SP38, DMKU-SP95	4
Moesziomyces parantarcticus	DMKU-RP39	1
Pseudozyma hubeiensis	DMKU-SP13, YE-21	2
Ustilago siamensis	DMKU-SE62, YE-86	2

#

Table S2 Antagonistic activities of antagonistic yeast strains against *L. theobromae* and *C. gloeosporiodes* by dual cultivation on PDA plates and activities of VOCs produced by these strains by double plate technique at 25°C for 3 and 14 days, respectively

Treatment	Fungal pathogens growth inhibition (%)[a]	
	Antagonistic activity	Activity of VOCs
T. indica DMKU-RP31 + *L. theobromae*	67.7±0.89a	51.3±7.54a
T. indica DMKU-RP35 + *L. theobromae*	67.9±4.40a	59.8±4.66a
Ps. hubeiensis YE-21 + *L. theobromae*	56.7±0.89b	34.4±4.34b
P. aspenensis DMKU-SP67 + *C. gloeosporiodes*	66.3±3.74	55.6±2.86

In the same column data followed by the different, same, and overlapping lower-case letters means significantly different, and no significantly different of their overlapping to Duncan's multiple range test at $p \leq 0.05$. Each result presents the mean ± standard derivation from three replicates
[a]Inhibition (%) = (radius of control fungal colony – radius of fungal colony grows with yeast) × 100/radius of control fungal colony
